# A new species of *Ripipteryx* from Belize with a key to the species of the Scrofulosa Group (Orthoptera, Ripipterygidae)

**DOI:** 10.3897/zookeys.169.2531

**Published:** 2012-02-10

**Authors:** Sam W. Heads, Steven J. Taylor

**Affiliations:** 1 Illinois Natural History Survey, Prairie Research Institute, University of Illinois at Urbana-Champaign, 1816 South Oak Street, Champaign, Illinois 61820-6960, USA

**Keywords:** Orthoptera, Caelifera, Tridactyloidea, Ripipterygidae, *Ripipteryx*, new species, Mesoamerica

## Abstract

A new species of the genus *Ripipteryx* (Orthoptera: Tridactyloidea: Ripipterygidae) from the Toledo District of southern Belize is described and illustrated. *Ripipteryx mopana* **sp. n.** is placed in the Scrofulosa Group based on its elaborately ornamented frons and is readily distinguished from its congeners by the fusion of the superior and inferior frontal folds to form a nasiform median process, the epiproct with both anterior and posterior margins emarginate, the subgenital plate with distinct lateroapical depressions either side of the median line, the basal plate of the phallus strongly bilobed apically, and the development of well-demarcated denticular lobes in the dorsal endophallic valves. A preliminary key to the species of the Scrofulosa Group is provided.

## Introduction

Neotropical tridactyloids are both diminutive and cryptic, and being collected only rarely, are also underrepresented in collections. The tridactyloid fauna of Central America in particular is extremely diverse and yet simultaneously poorly documented. Indeed, only 19 tridactyloid species are recorded from Mesoamerica compared to over 100 known from South America (Otte 1997; Günther 1980; Eades et al. 2011). Work on Mesoamerican tridactyloids began with Saussure’s (1859) description of *Ripipteryx mexicana* from Oaxaca, Mexico and continued with his contribution to *Biologia Centrali-Americana* (Saussure 1896) in which he described 17 additional species. These taxa were subsequently revised by Günther (1969, 1975–1977, 1989) who added several species to the fauna and synonymized others. Despite Günther’s work however, chronic under-sampling in the region means that very little is known about tridactyloid diversity in Mesoamerica and even less about their biology.

The genus *Ripipteryx* Newman, 1834 is exclusively Neotropical, with some 44 species distributed throughout South and Central America (Günther 1969, 1980; Heads 2010). The majority of *Ripipteryx* species known from Mesoamerica belong to the Scrofulosa Group, a presumably monophyletic group comprised of small, variegated species characterized by their peculiar and elaborately ornamented frons and tuberculate or denticulate dorsal endophallic valvulae (Heads 2010). Here, we provide for the first time, a key to the Scrofulosa Group and describe a distinctive new species as the first record of the family Ripipterygidae from Belize.

## Material and methods

The holotype is deposited in the Entomology Collection of the Illinois Natural History Survey (INHS), Prairie Research Institute, University of Illinois, and was studied using an Olympus SZX12 zoom stereomicroscope with 1× and 2× objectives. Drawings were produced with the aid of a *camera lucida*. Photomicrographs were made using a digital SLR camera and 65 mm macro lens. To examine the terminalia and phallic complex, the abdomen was removed using Vannas’ scissors and cleared in warm 10% KOH. The phallus was then dissected and subsequently stored together with the terminalia under glycerin in a glass microvial pinned beneath the specimen. Terminology generally follows that of Heads (2010) with modifications concerning structures associated with the highly modified male paraproct. In most tridactyloids, the paraproct bears two distinctive processes: [1] a well-sclerotized proximal hook-like structure, herein termed the *uncus* (“Hakensklerit” of Günther 1969); and [2] an elongate, cercus-like structure, herein termed the *brachium* (“Paraproctfortsatz” of Günther 1969).

## Systematics

### Genus Ripipteryx Newman, 1834. Scrofulosa Group sensu Heads, 2010

#### Ripipteryx mopana

Heads & Taylor sp. n.

urn:lsid:zoobank.org:act:93A6697D-C161-4275-B1D7-6FC0F9D3245F

urn:lsid:orthoptera.speciesfile.org:TaxonName:73795

http://species-id.net/wiki/Ripipteryx_mopana

Figs 1–5

##### Diagnosis.

 The new species is readily separated from other small, variegated *Ripipteryx* by the elaborately ornamented frons characteristic of Scrofulosa Group species. From other members of the Scrofulosa Group the new species is distinguished by [1] fusion of the superior and inferior frontal folds forming a nasiform median process; [2] the emarginated anterior and posterior margins of the epiproct; [3] the subgenital plate with distinct lateroapical depressions either side of the median line; [4] the strongly bilobed apex of the basal plate of phallus; and [5] the presence of well-demarcated denticular lobes in the dorsal endophallic valves.

##### Description.

**Figures 1–5.**
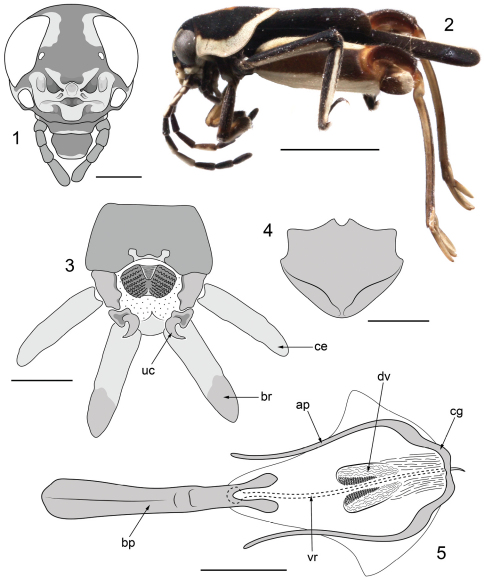
Holotype ♂ of*Ripipteryx mopana* Heads & Taylor, sp. n. **1** frontal view of head capsule with antennae omitted (scale bar 0.5 mm) **2** lateral habitus (scale bar 2.0 mm) **3** dorsal view of terminalia with setae omitted for clarity (scale bar 0.25 mm) **4** ventral view of subgenital plate with setae omitted for clarity (scale bar 0.25 mm) **5** dorsal view of phallic complex (scale bar 0.25 mm). Abbreviations: **ap** apodemes of cingulum; **bp** basal plate; **br** brachium; **ce** cercus; **cg** cingulum; **dv** dorsal valve; **uc** uncus; **vr** virga.

*Male*: Body form small (length 4.54 mm from frons to apex of subgenital plate) and compact with coloration highly variegated (Figs 1–2). Vertex largely black, with crescent-shaped pale cream patches circumscribing the anterodorsal margins of the compound eyes. Interocular distance 0.73 mm. Compound eyes broadly subovoid, 0.86 mm high. Lateral ocelli very small, situated very close to the medial margin of the compound eyes. Median ocellus absent. Frons largely pale cream fringed with reddish brown and bearing numerous elaborate folds and lobes; comprising a central nasiform process formed through fusion of the superior and inferior folds, flanked by deep, sinuous furrows themselves bordered by broad ridges and lobes; frontoclypeal lobe present (Fig. 1). Antennae ten segmented, moniliform, inserted directly beneath the compound eyes. Scape twice as long as pedicel; flagellomeres densely pubescent and wider apically than at their base. Scape, pedicel and flagellomeres 1 and 2 pale cream dorsally and black ventrally; flagellomere 3 almost entirely black; flagellomere 4 with triangular-shaped pale cream patch dorsally and black ventrally; flagellomere 5 almost entirely pale cream; remaining flagellomeres entirely black. Pronotum somewhat tectate anteriorly (Fig. 2), 1.77 mm long, broadly rounded posteriorly; black with broad, pale cream lateral and posterior margins and a prominent orange-brown median patch dorsally that is obovate anteriorly and rhombiform posteriorly. Tegmen entirely black, 2.38 mm long. Hind wing remigium entirely black; posterior fan cream. Profemora 1.18 mm long, black to dark brown dorsally and pale brown to cream ventrally. Protibiae claviform and largely black with a pale cream longitudinal stripe. Mesofemora 1.91 mm long, subquadrate in section, black dorsally and pale cream ventrally. Mesotibiae black with a prominent pale cream longitudinal stripe along the dorsolateral margin. Metafemora large and robust, 3.28 mm long, reddish brown medially with broad pale cream bands dorsally and ventrally; geniculae well-developed, dark reddish brown with pale cream apices. Metatibiae 3.14 mm long, pale yellowish brown with prominent darker dorsal carinae; apical metatibial spurs blade-like with prominent apical hooks, more than twice as long as subapical spurs. Metatarsus sublanceolate, 0.66 mm long, marginally shorter than the apical metatibial spurs. Posterior margin of abdominal tergite 10 broadly emarginate with prominently bilobed membranous median region (Fig. 3). Epiproct with large, densely reticulate lateral lobes and emarginate anterior and posterior margins. Cerci fusiform, bearing numerous long and evenly spaced setae. Paraprocts with large, well-sclerotized and strongly hooked uncuses and robust, apically thickened brachia bearing numerous strong ventroapical setae; brachia only marginally longer than cerci (Fig. 3). Subgenital plate broadly rounded with prominent lateroapical depressions either side of the median line; densely pubescent apically (Fig. 4). Phallus with basal plate strongly bilobed apically; cingulum broad and furcate, thickened laterally and bearing elongate, gently curved apodemes; dorsal valves of the endophallus forming flexible lobes armed with numerous denticles; virga filiform with an uncinate basal articulating process (Fig. 5).

*Female*: Unknown.

##### Holotype.

 ♂: Belize, Toledo District, hand collected on shore of Rio Grande at night, approx. 2 hrs after sunset, 28.1 km NNW of Punta Gorda, 16.31739°N, 88.93442°W, 15 April 2011, sjt11-016, coll. S. J. Taylor, sample # 231, specimen # 0338 (INHS).

##### Etymology.

 The specific epithet honors the Mopan, a Mayan people that live primarily in the southern part of Belize where the new species was collected. There is considerable ethno-historic and toponymic evidence to suggest that the Mopan have lived in this region since before the Spanish conquest (Jones 1998; Wainwright 2009). The Mopan people are recognized by their eponymous language (a form of Yucatec Mayan), spoken by 11,800 people in Belize and Guatemala (Lewis 2009). The gender of the epithet is feminine.

##### Remarks.

 Ordinarily, we would hesitate to describe a new species based on a single specimen. However, given the number of robust morphological apomorphies there can be no doubt that *Ripipteryx mopana* is a distinct species. Within the Scrofulosa Group, *Ripipteryx mopana* is most similar to *Ripipteryx biolleyi* Saussure, 1896 sharing with this species the loss of the median ocellus and the distinctive nasiform frontal process. The nasiform process in *Ripipteryx biolleyi* is formed by the upturned apex of the inferior fold strongly overlapping that of the superior fold. *Ripipteryx mopana* differs in that the apex of the inferior fold is completely fused to the underlying superior fold (Fig. 1). The frontal ornament of *Ripipteryx mopana* further differs from that of *Ripipteryx biolleyi* in the presence of carinulated pits on the lateral lobes of the inferior fold and deep, sinuous furrows (rather than ovoid cavities as in *Ripipteryx biolleyi*) flanking the nasiform process. Both species possess a furcate cingulum with long, slender apodemes, though the apex of the basal plate is strongly bifurcated in *Ripipteryx mopana* and undivided in *Ripipteryx biolleyi*. Together, *Ripipteryx biolleyi* and *Ripipteryx mopana* appear to be most closely related to *Ripipteryx saltator* Saussure, 1896 and *Ripipteryx saussurei* Günther, 1969 sharing with these species a deep invagination of the inferior fold above the frontoclypeal lobe and the development of well-sclerotized denticles in the dorsal valves of the endophallus. These denticles are directed posteriorly and arranged in rows along valvular axial lobes, which are particularly well developed in *Ripipteryx mopana* (Fig. 5). Denticular lobes are not present in *Ripipteryx mediolineata* Saussure, 1896, *Ripipteryx mexicana* Saussure, 1859, *Ripipteryx scrofulosa* Günther, 1969 and *Ripipteryx tricolor* Saussure, 1896 all of which instead possess rows of weakly sclerotized, tubercle-like rugosities (Günther 1969). Of these species, *Ripipteryx mediolineata* and *Ripipteryx scrofulosa* are apparently the most primitive of the group having the frontal folds poorly developed and lacking a frontoclypeal lobe.

### Preliminary key to species of the Scrofulosa Group

**Table:** 

1	Frontal folds poorly-developed; frontoclypeal lobe absent; uncus reduced with retrograde apex; brachium long, about twice the length of the cercus	2
–	Frontal folds well-developed; frontoclypeal lobe present; uncus large with either retrograde or dorsolaterally directed apex; brachium almost equal in length to slightly longer than cercus	3
2	Inferior frontal fold pale cream or white with two small black spots; pronotum black with broad yellowish white margins; brachium strongly claviform	*Ripipteryx scrofulosa* Günther
–	Inferior frontal fold entirely pale cream or white, lacking black spots; pronotum black with broad yellowish white margins and a distinctive median yellow stripe with black spots in anterior half; brachium fusiform	*Ripipteryx mediolineata* Günther
3	Inferior frontal fold with shallow depression above the frontoclypeal lobe; dorsal valves of endophallus with weakly sclerotized tubercle-like rugosities	4
–	Inferior frontal fold with deep invagination above the frontoclypeal lobe; dorsal valves of endophallus with more or less developed lobes bearing well-sclerotized rows of posteriorly directed denticles	5
4	Frontal depression lenticular and flanked by two small black spots; posterior margin of abdominal tergum 10 with median membranous region unilobate	*Ripipteryx tricolor* Saussure
–	Frontal depression ovoid and lacking black spots; posterior margin of abdominal tergum 10 with median membranous region bilobed	*Ripipteryx mexicana* Saussure
5	Median ocellus nascent or entirely lost; lateral lobes of inferior frontal fold with prominent rounded callosities or pits; frontoclypeal lobe well-developed	6
–	Median ocellus present; lateral lobes of inferior frontal fold with a shallow longitudinal sulcus; frontoclypeal lobe weakly developed (Fig. 6)	*Ripipteryx saussurei* Günther
6	Median ocellus entirely lost; apices of superior and inferior folds strongly overlapped or fused forming a nasiform process; subgenital plate broadly rounded	7
–	Median ocellus nascent; apices of superior and inferior folds closely approximated but not overlapping (Fig. 7); subgenital plate paraboliform	*Ripipteryx saltator* Saussure
7	Nasiform process formed from strongly overlapping apex of inferior frontal fold and flanked by deep, ovoid cavities; lateral lobes of inferior frontal fold with two swollen callosities, the dorsalmost at least twice as large as the ventral (Fig. 8); apex of basal plate undivided; dorsal valves of endophallus with poorly-developed denticular lobes	*Ripipteryx biolleyi* Saussure
–	Nasiform process formed from fused apices of the superior and inferior frontal folds and flanked by deep sinuous furrows; lateral lobes of inferior frontal folds with large outer and smaller inner pits each bordered by very weak carinulae; apex of basal plate strongly bifurcate; dorsal valves of endophallus with well-developed denticular lobes	*Ripipteryx mopana* Heads & Taylor, sp. n.

**Figures 6–8.**
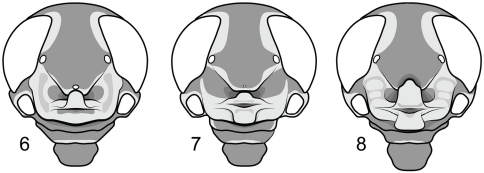
Head capsules of representative Scrofulosa Group species. **6** *Ripipteryx saussurei* Günther (Mexico); **7** *Ripipteryx saltator* Saussure (Costa Rica) **8** *Ripipteryx biolleyi* Saussure (Costa Rica).

## Supplementary Material

XML Treatment for Ripipteryx mopana
